# Photoactivation: The Light-Driven Assembly of the Water Oxidation Complex of Photosystem II

**DOI:** 10.3389/fpls.2016.00578

**Published:** 2016-05-03

**Authors:** Han Bao, Robert L. Burnap

**Affiliations:** Department of Microbiology and Molecular Genetics, Oklahoma State UniversityStillwater, OK, USA

**Keywords:** photosystem II, water oxidation, manganese, oxygen evolution, photoactivation, EPR, oxo bridge

## Abstract

Photosynthetic water oxidation is catalyzed by the Mn_4_CaO_5_ cluster of photosystem II. The assembly of the Mn_4_O_5_Ca requires light and involves a sequential process called photoactivation. This process harnesses the charge-separation of the photochemical reaction center and the coordination environment provided by the amino acid side chains of the protein to oxidize and organize the incoming manganese ions to form the oxo-bridged metal cluster capable of H_2_O-oxidation. Although most aspects of this assembly process remain poorly understood, recent advances in the elucidation of the crystal structure of the fully assembled cyanobacterial PSII complex help in the interpretation of the rich history of experiments designed to understand this process. Moreover, recent insights on the structure and stability of the constituent ions of the Mn_4_CaO_5_ cluster may guide future experiments. Here we consider the literature and suggest possible models of assembly including one involving single Mn^2+^ oxidation site for all Mn but requiring ion relocation.

## Introduction

A decline in the photosynthetic activity of oxygenic photosynthetic organisms due to light stress has been described as photoinhibition (Björkman, 1981; Osmond, 1981; Powles and Björkman, 1981; Ohad et al., 1984). The primary damage occurs within the reaction center of Photosystem II (PSII). It is distinct from the concurrent oxidative damage to the machinery of protein synthesis, which compounds the problem since *de novo* protein synthesis is necessary for the replacement of damaged PSII proteins (Adir et al., 2003; Lupínková and Komenda, 2004; Nishiyama et al., 2004; Edelman and Mattoo, 2008). The precise mechanism of PSII photoinhibition *in vivo* remains under debate (Adir et al., 2003; Edelman and Mattoo, 2008; Vass and Cser, 2009). Despite this uncertainty, it is evident that the D1 reaction center protein is the primary target for photodamage and this leads to an increased turnover rate of D1, in comparison to other PSII proteins, upon exposure to high light intensities (Ohad et al., 1984). To cope with light stress, all oxygenic photosynthetic organisms have developed protective mechanisms both to minimize the effects of exposure to excess light and to efficiently repair the damage when it occurs. Overall, the efficiency of photosynthetic electron transfer decreases markedly only when the rate of damage exceeds the rate of repair. A crucial phase of the *de novo* biogenesis of PSII, as well as the damage repair process, is the assembly of the Mn_4_CaO_5_ complex. This involves the oxidative assembly of Mn^2+^ and Ca^2+^ ions into the coordination environment of the PSII water-oxidation complex (WOC) in a light-driven process called photoactivation (for previous reviews, see Ono, 2001; Burnap, 2004; Dismukes et al., 2005).

## PSII damage and D1 replacement

### Replacement of damaged D1

**Figure 1:**
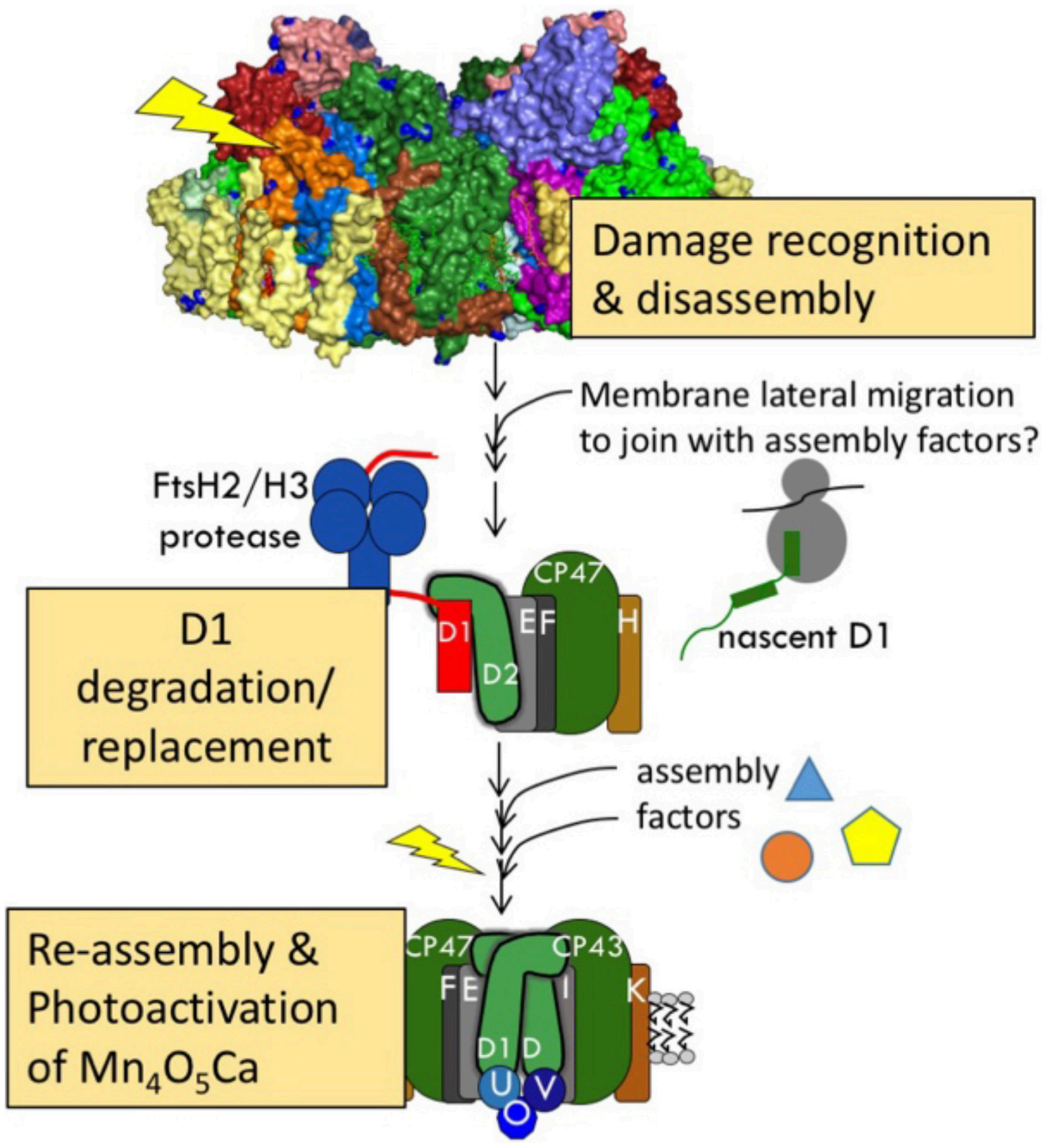
**Schematic repair pathway for photodamaged PSII**. The process can be divided into the three main phases: (1) damage recognition and partial disassembly of photodamage PSII complexes, (2) D1 degradation and replacement, and (3) reassembly of the subunits and light-driven assembly (photoactivation) of the Mn_4_O_5_Ca metal cluster.

The entire process of PSII damage-repair cycle can be described as follows: (i) damage occurring to PSII, (ii) signaling of this damage, (iii) monomerization of PSII dimer and partial disassembly of PSII monomer, (iv) degradation of D1 and insertion of a newly synthesized D1 into PSII sub-complex, and (v) reassembly of holoenzyme and photoactivation of the Mn_4_CaO_5_ cluster (Aro et al., 1993; Koivuniemi et al., 1995; Nixon et al., 2005; Figure 1). We briefly outline some features of the overall PSII assembly and repair process to place the assembly of the Mn_4_CaO_5_ cluster in context. For more comprehensive information the reader is advised to examine several recent review articles (Nixon et al., 2010; Becker et al., 2011; Nickelsen and Rengstl, 2013; Heinz et al., 2016).

Monomerization of dimeric PSII has been suggested to result from the detachment or rearrangement of PsbO, one of three luminal extrinsic subunits of PSII (Nixon et al., 2010). The basis of this assessment is the failure to accumulate dimeric PSII in a mutant of *Synechocystis* sp. PCC 6803 (hereafter *Synechocystis* 6803) lacking PsbO (Komenda et al., 2010). In plants and green algae, it has also been proposed that PSII core phosphorylation might trigger disassembly of PSII dimer to form monomer by acting alone or in conjunction with PsbO (Puthiyaveetil and Kirchhoff, 2013). Detachment of CP43 from PSII monomer leads to the formation of so-called RC47 complex which is a pivotal sub-complex for further replacement of damaged D1 during PSII repair (Komenda et al., 2005). Given the fact that PsbO functions as PSII manganese-stabilizing protein and CP43 participates with D1 in ligating the Mn_4_CaO_5_ cluster, it is conceivable that photodamage to Mn_4_CaO_5_ cluster might cause the detachment of these two subunits. It is also interesting to note that the assembly and disassembly of the Mn_4_O_5_Ca regulates the coupling of the phycobilisome to the cyanobacterial PSII reaction center such that centers without an intact metal cluster are not efficiently coupled with respect to energy transfer from the phycobilisome (Hwang et al., 2008).

Radioactive pulse-chase experiments (Komenda and Barber, 1995) showed that translation inhibitors slow D1 degradation, suggesting that D1 degradation and new D1 synthesis are synchronized. Increased turnover of D1 could be a generalized response to damage-promoting light conditions, with all D1 copies prone to increased probability of replacement or there could be a specific targeting mechanism that replaces only damaged D1 copies. Intuitively, a targeting mechanism seems more likely. However, despite good circumstantial evidence, direct evidence for the specific targeting of PSII centers with damaged D1 has not been obtained, mainly because it is technically difficult to separately track damaged and undamaged forms of D1 through the replacement process. Recently, targeting has been inferred from experiments where cells are allowed to express two alternative forms of the D1 protein in the same cell, with one wild-type form and the other a light-sensitive form. The analysis indicates that only the light-sensitive version of D1 and not the wild-type version is turned over very rapidly (Nagarajan and Burnap, 2014).

FtsH proteases play an important role in degradation of damaged D1 during PSII repair (Mann et al., 2000; Bailey et al., 2002; Silva et al., 2003). Mutants lacking FtsH proteases display impaired D1 degradation and thus accumulate damaged D1 (Bailey et al., 2002; Silva et al., 2003; Komenda et al., 2006; Kato et al., 2009). Additionally, the AAA-type protease, FtsH is crucial for the degradation of D1 protein (Silva et al., 2003; Nixon et al., 2005). Without it efficient repair ceases. How newly synthesized D1 subunit is integrated into the RC47 sub-complex is still a matter of debate. Studies in chloroplasts have led to the conclusion that D1 replacement occurs co-translationally (Zhang et al., 1999, 2000). Following initiation of *psbA* mRNA translation, nascent D1 protein is targeted to the thylakoid membrane by the chloroplast signal recognition particle (cpSRP54) and then released after interacting with a putative SRP receptor (Zhang and Aro, 2002). It was demonstrated that cpSRP54 can be efficiently crosslinked to nascent D1 chains that are still attached to ribosomes (Nilsson et al., 1999; Nilsson and van Wijk, 2002). Polypeptide chain elongation of the docked complex results in precursor D1 (pD1, see below) insertion into the thylakoid translocation channel (cpSecY) (Zhang et al., 2001). During translocation, the transmembrane domains of nascent pD1 appear to interact with existing PSII sub-complexes containing D2, PsbI, and cytochrome b_559_, CP47, but lacking CP43 (van Wijk et al., 1996, 1997; Zhang and Aro, 2002) and subsequently incorporate into PSII complex. Pulse-labeling studies indicated that this association already exists before the synthesis of the pD1 protein is complete (Zhang et al., 1999, 2000).

There is still uncertainty about where the repair of damaged PSII takes place and it is worth noting that PSII assembly for repair and PSII *de novo* assembly appear to involve distinct PSII assembly pathways (reviewed in Heinz et al., 2016). Regions of connection between the plasma membrane and thylakoid membrane appear to be sites of PSII assembly (Klinkert et al., 2004; Schottkowski et al., 2009; Nickelsen et al., 2011; Stengel et al., 2012; Heinz et al., 2016). These studies have led to the suggestion that regions of the thylakoid membrane are differentiated by being specifically enriched in assembly proteins, which are designated PratA-defined membranes (PDMs) being especially relevant to the photoassembly of the Mn_4_CaO_5_ as discussed below. The idea of localized region of the thylakoid membrane enriched in assembly factors fits with the report that FtsH proteins are localized in thylakoids (Komenda et al., 2006; Krynická et al., 2014) in distinct patches that are less enriched in chlorophyll (Sacharz et al., 2015). Thus, from an ultrastructural perspective, a reasonable working hypothesis is that the repair processes, including photoactivation, are located in discreet regions of the thylakoid enriched in the factors facilitating reassembly.

### Processing of the D1 carboxy terminus

D1 protein is synthesized in a precursor form (pD1) with a carboxyl-terminal extension (C-terminal) whose length and sequence vary among different organisms (Diner et al., 1988b; Seibert et al., 1989; Nixon et al., 1992; Anbudurai et al., 1994; Shestakov et al., 1994; Ivleva et al., 2000; Zhang and Aro, 2002). The pD1 protein is subsequently cleaved on the carboxyl side of residue Ala344, resulting in the removal of the extension (Nixon and Diner, 1992; Nixon et al., 1992), which is carried out by carboxy terminal protease (CtpA), which is dedicated to this post-translational processing (Diner et al., 1988a; Seibert et al., 1989; Nixon et al., 1992; Taguchi et al., 1995; Trost et al., 1997; Ivleva et al., 2000). In plants, an extension consisting of 9 residues is cleaved in a single proteolytic step, whereas in *Synechocystis* 6803 a 16 amino acid extension is removed in two steps (Komenda et al., 2007; Satoh and Yamamoto, 2007). Although the extension is not essential for assembly of functional PSII complex (Nixon et al., 1992; Satoh and Yamamoto, 2007), it is required for optimal photosynthetic performance implying that it might plays an important role in PSII repair (Diner, 2001). For example, *Synechocystis* mutants lacking the C-terminal extension exhibit decreased fitness and are more susceptible to photodamage (Ivleva et al., 2000; Kuviková et al., 2005). D1 maturation is a prerequisite for assembly of the Mn_4_CaO_5_ cluster (Diner et al., 1991; Nixon et al., 1992) and binding of the PSII extrinsic proteins (Roose and Pakrasi, 2004), thus is essential for oxygen evolution activity (Taylor et al., 1988). The extension must be cleaved before the Mn_4_CaO_5_ cluster can be functionally assembled (Nixon et al., 1992; Anbudurai et al., 1994; Komenda et al., 2007), suggesting the C-terminus of the mature D1 polypeptide is involved in assembly of the Mn_4_CaO_5_ cluster. In recent X-ray structures (Umena et al., 2011), Ala344 is shown to coordinate the Mn(2) and the Ca atom of Mn_4_CaO_5_ through its backbone α-carboxyl moiety. These assignments are consistent with mutational analysis that had originally led to this suggestion (Diner et al., 1991; Nixon et al., 1992)

### Accessory proteins for PSII assembly and repair

Numerous accessory proteins are being discovered to have roles in the assembly, maturation and repair of the PSII complex (Shestakov et al., 1994; Inagaki et al., 2001; Yamamoto, 2001; Kashino et al., 2002; Silva et al., 2003; Roose and Pakrasi, 2004; Keren et al., 2005; Chen et al., 2006; Komenda et al., 2006; Nowaczyk et al., 2006; Park et al., 2007). All full accounting of these is beyond the scope of this review and for the most recent summary of the numerous assembly factors the reader should consult (Heinz et al., 2016). Biochemical approaches (e.g., Nowaczyk et al., 2006; Mamedov et al., 2007) and genetic analyses (e.g., Klinkert et al., 2004; Liu et al., 2011b), have led to the identification of proteins facilitating the assembly of PSII that could be of specific relevance to the process of photoactivation, most notably, PratA and Psb27. Deletion of *pratA* results in a dramatic decrease in the accumulation of PSII in *Synechocystis* and a defect in the processing of the D1 C-terminus by CtpA. Moreover, PratA interacts with the D1 C-terminus and may bind Mn^2+^ possibly facilitating the assembly of the Mn_4_O_5_Ca (Klinkert et al., 2004; Schottkowski et al., 2009). Psb27 is found to bind to forms of the PSII complex thought to represent assembly and/or disassembly intermediates (Roose and Pakrasi, 2004; Nowaczyk et al., 2006; Mamedov et al., 2007; Liu et al., 2011a,b) and deletion of the protein affects photoactivation of the complex (Roose and Pakrasi, 2007). Thus, Psb27 and PratA are especially good candidates for facilitating photoactivation of the Mn_4_CaO_5_. Indeed, there is good reason to believe that the published *in vitro* assembly experiments are missing assembly cofactors, which may explain why the yield of active PSII centers produced by *in vitro* photoactivation of Mn_4_CaO_5_ clusters by biochemical methods is invariably lower than intact cells as discussed below.

## Mechanism of photoactivation

### Coordinating residues of Mn_4_CaO_5_ cluster

**Figure 2:**
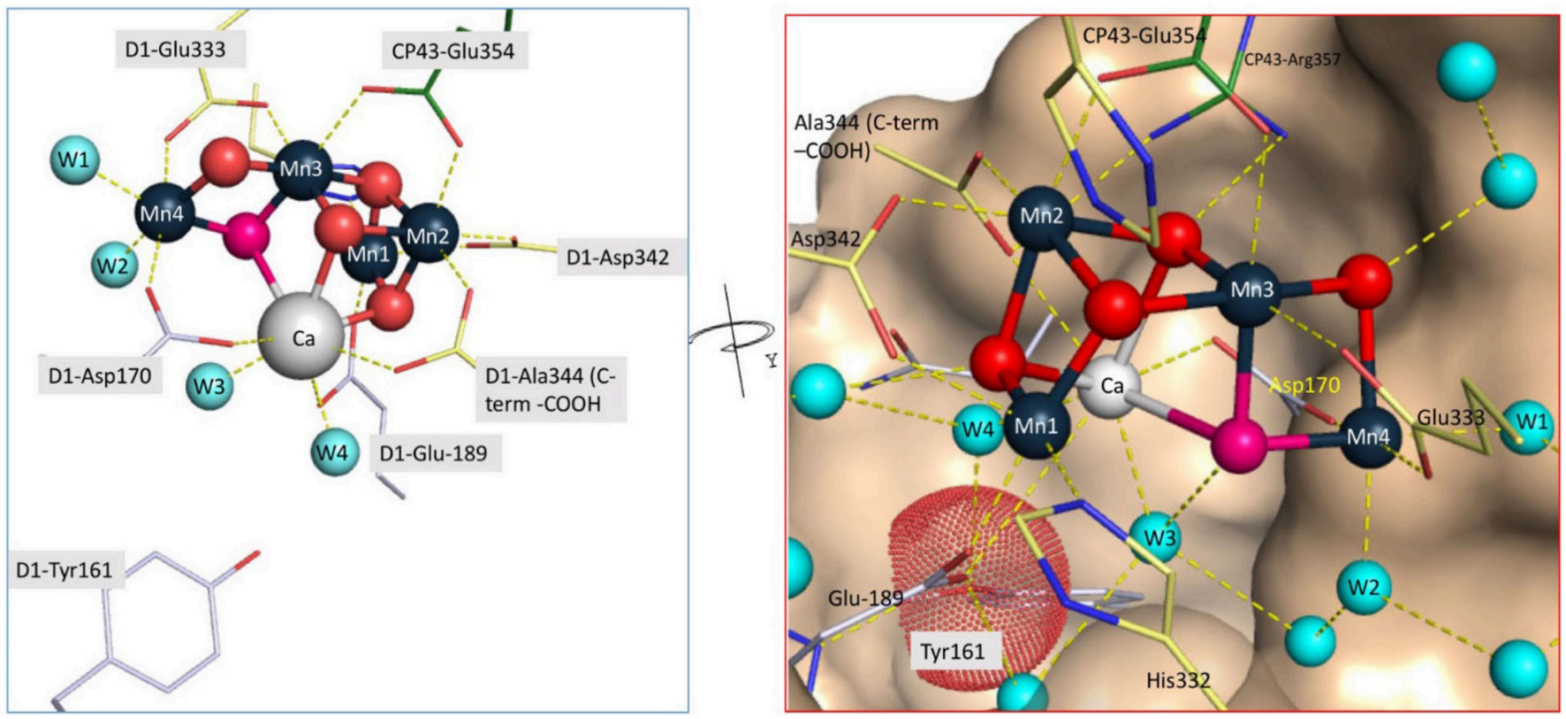
**Coordination environment of the assembled Mn_**4**_CaO_**5**_ H_**2**_O-oxidation complex of PSII**. The high affinity site of Mn^2+^ binding and photooxidation during the initial phase of the assembly process minimally involves D1-Asparate170 (Nixon and Diner, 1992; Campbell et al., 2000) located in the vicinity of Mn4 in the final complex. The initial state of the complex for photoassembly appears to involve the binding of one Mn^2+^ at the high affinity site (Ono and Mino, 1999) together with one Ca^2+^ ion that modulates the ligand environment of the Mn^2+^ possibly via the formation of a bridging water or hydroxide, although the presence of the Ca^2+^ does not appreciably change the binding affinity of the Mn^2+^ at the high affinity site (Tyryshkin et al., 2006). The C-terminal polypeptide backbone carboxylate of D1-Alanine344, which is available only following proteolytic cleavage of the precursor form of the D1 protein (pD1), is also critical for the assembly process, although it too does not markedly alter the binding of Mn^2+^ at the high affinity site (Nixon et al., 1992; Cohen et al., 2007). Figures developed upon 3D coordinates (PDB 4UB6) of the published X-ray diffraction model (Umena et al., 2011).

According to 1.9 Å PSII crystal structure (Umena et al., 2011), Mn_4_O_5_Ca cluster coordinated by one nitrogen ligand from D1-His332 and six carboxylate ligands from D1-Asp170, D1-Glu189, D1-Glu333, D1-Asp342, D-Ala344, CP43-Glu354 (Figure 2). Three of them, D1-Glu333, D1-Asp342, and CP43-Glu354, form μ–carboxylate bridges between Mn (Mn(1)–Mn(2) (Asp342), Mn(2)–Mn(3) (CP43-Glu354), and Mn(3)–Mn(4) (Glu333)). D1-Asp170 and the C-terminal carboxylate group of D1-Ala344 bridge Ca with Mn(4) and Mn(2), respectively. The Mn(4) has been referred to as the “dangler manganese” (Peloquin et al., 2000) because it is located outside the semi-cubic cluster formed by the other four metals of the cluster, Ca, Mn(1), Mn(2), and Mn(3). Both D1-Glu189 and D1-His332 serve as monodentate ligands to Mn(1). The D1-Asp170 plays an especially crucial role during the assembly process since it helps form the so-called “high affinity site” involved in the initial photooxidation of Mn^2+^ (Nixon and Diner, 1992).

### Two-quantum model of photoactivation

**Figure 3:**
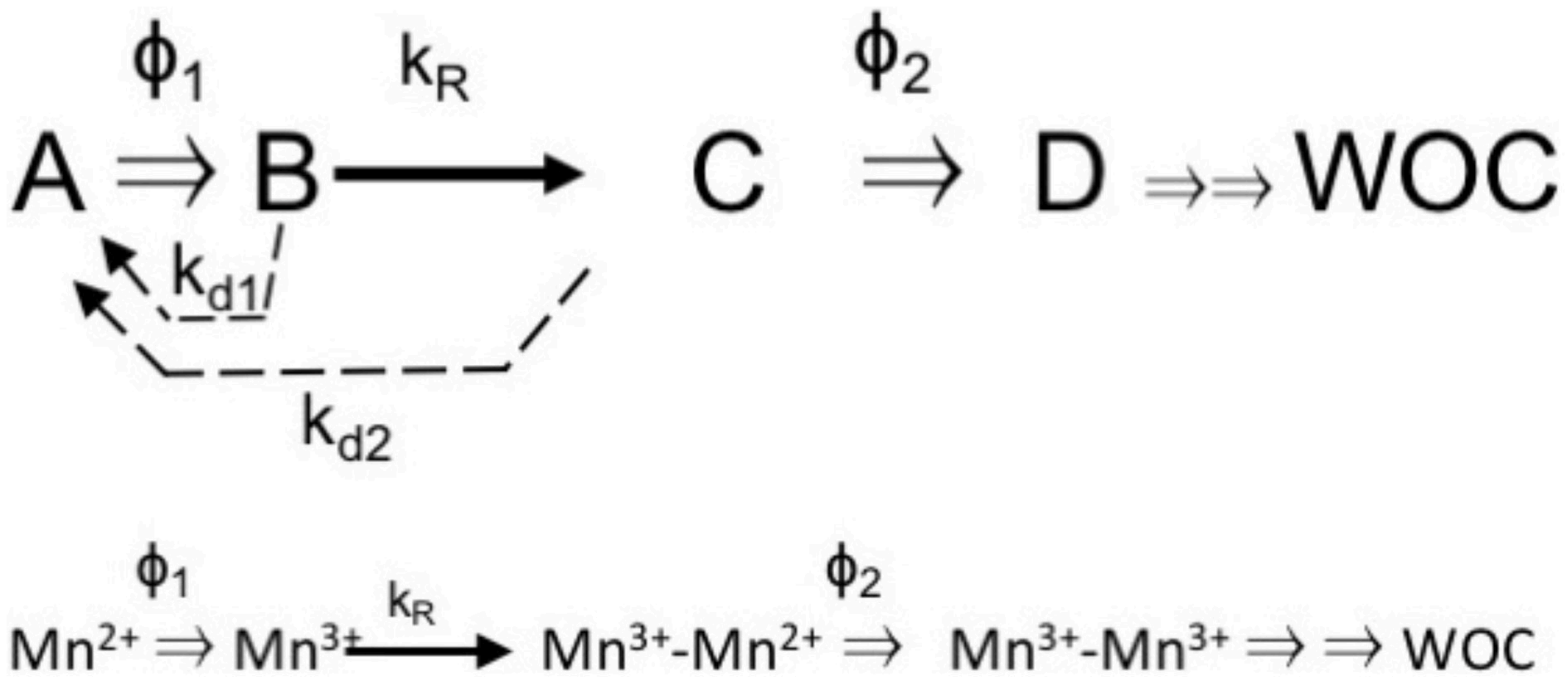
**Kinetic scheme of basic two-quantum mechanism**. Double arrows indicate light-activated processes with the quantum efficiencies Φ_1_ and Φ_2_ representing the first and second photooxidative events in the assembly sequence, k_*R*_ representing the still ill-defined “dark” rearrangement, and k_*D*1_, k_*D*2_ representing the decay of intermediates. After the initial two Mn are photoligated, subsequent Mn appear to be added with high quantum yield.

The assembly of the metals of the Mn_4_O_5_Ca requires light to induce charge separation to oxidize and strongly bind the Mn ions. It is important to note that the assembly is an oxidative process that involves removal of electrons from the Mn ions and the formation of oxo-bridges between the metals of the cluster with the bridging oxygen atoms (shown in red, Figure 2) derived from water molecular coordinated to the metal ions. The oxidative assembly utilizes the same light-driven charge separation events within the photochemical reaction center that subsequently drive photosynthetic electron transfer in the fully functional enzyme. Apart from the definition of the Mn-binding site characteristics and some very well-defined kinetic features that govern the development of H_2_O-oxidation activity, photoactivation remains poorly understood. The quantum efficiency of photoactivation is very low, typically in the range of ~1%, which is much lower than for photosynthetic water oxidation in the assembled PSII (>90%) even in intact systems (Cheniae and Martin, 1971a,b; Cheniae and Martin, 1972; Radmer and Cheniae, 1971; Ono and Inoue, 1982, 1983). The kinetic model of photoactivation, termed as “two-quantum series model” (Radmer and Cheniae, 1971), was originally observed during photoactivation as a function of either light intensity or flash interval using fixed numbers of Xe light flashes (Cheniae and Martin, 1971a,b; Cheniae and Martin, 1972; Radmer and Cheniae, 1971). These pioneering studies showed that the quantum efficiency for photoactivation is low at low light intensities, reached a maximum at intermediate intensities, and were again low at high light intensities. Equivalently, the quantum efficiency is low when saturating, single turnover flashes are given at long intervals, maximum at intermediate flash frequencies (~1 per second), and were again low when the flashes are given with short intervals between flashes. From these features, Cheniae derived a minimal model, the so-called two-quantum model that postulated the light-induced Mn assembly with at least one unstable chemical intermediate as depicted in Figure 3. The first photoevent involves the high quantum yield photooxidation of a single Mn^2+^ to Mn^3+^ ion (Ono and Mino, 1999) at the unique high affinity Mn-binding site (see below). The resultant Mn^3+^ species (B) can spontaneously convert to C in the dark with a 100–150 mshalf-time, with a kinetic constant designated k_*R*_ in the scheme in Figure 3. A second quantum of light must be absorbed to convert the nascent complex into the first stable intermediate D as shown in Figure 3 as C⇒D. The formation of a labile intermediate, t_1∕2_ ~1–2 s, accounted for the optimum in light intensity or, alternatively, flash frequency, utilized for the assembly process. Photoactivation using saturating single turnover flashes is optimal with flash spacing of ~1 s, which is enough time to allow the dark rearrangement to occur (k_*R*_), but short enough to minimize the decay of the intermediate(s). If, however, the flash interval is too long, the second flash is not in time to trap forward progress and the reactants decay (k_*D*1_, K_*D*2_, Figure 3). The molecular nature of the process occurring during this dark rearrangement period (B→C) is not clear, and its understanding is key to understanding the overall molecular mechanism. After the initial two Mn are photoligated, subsequent Mn appear to be added with high quantum yield.

Of the many examples providing experimental support for the two quantum mechanism, perhaps the most striking are the experiments of Miyao, which showed a minimal two quantum requirement in an experiment where as few as five flashes restored nearly 20% of the maximal activity (Miyaotokutomi and Inoue, 1992). This amounts to several percent assembly per flash, which is remarkable given that the typical per flash yield is often on the order of 1% or even lower. That experiment and others also showed that the instability of the intermediates could be minimized by preventing the back-reaction of the electrons from the acceptor side of the PSII reaction center (Miyao and Inoue, 1991; Miyaotokutomi and Inoue, 1992). This also fits with another early result showing that the intermediates of assembly are highly sensitive to reductant (Ono and Inoue, 1987) and fits with the concept that the formation of state C (eligible for utilizing the second quantum) occurs with low frequency and/or once formed, the quantum yield of photooxidation of the second Mn^2+^ occurs with low quantum yield (also see Miller and Brudvig, 1989, for relevant model).

It has been speculated the rearrangement (k_*R*)_ involves a protein conformational change required for the binding and subsequent photooxidation of the second Mn^2+^ (Chen et al., 1995a; Ananyev and Dismukes, 1996b; Qian et al., 1999; Burnap, 2004). However, if (B → C) is indeed a protein structural change, then it is unlikely a large scale conformational rearrangement since carboxy terminal ligands are already close to high affinity site ligand D1-Asp170 during the first photooxidation (A⇒B) (Cohen et al., 2007). Also, whether the dark unstable intermediate is B or C (or both) remains unresolved. Given this uncertainty, Figure 3 shows both decays are possible (k_*D*1_ and k_*D*2_) (Miller and Brudvig, 1989). The development of a highly sensitive and fast Clark-type oxygen electrode (Ananyev and Dismukes, 1996b) led to the assignment of additional photoactivation intermediates and has provided alternative parameter estimates for the kinetic components (Ananyev and Dismukes, 1996a,b, 1997; Zaltsman et al., 1997; Baranov et al., 2000, 2004). At the same time, the use of this apparatus makes comparisons difficult because to the different illumination regimes. Most of the original experiments utilized single turnover Xe flashes for actinic illumination. In contrast, the photoactivation studies using the fast Clark-type oxygen electrode employed 30 ms red LED pulses promote optimum yields of assembled center (Ananyev and Dismukes, 1996b). This relatively long duration of the LED light pulses allows greater mixing of different assembly states because of the possibility of having multiple “hits” per center per pulse. That said, the 30 ms duration of the pulse is relatively short with respect to the t_1∕2_ ~150 ms of the B → C rearrangement and therefore the majority of those centers in the initial state that were excited (i.e., those undergoing A⇒B), will not be ready to utilize the second quantum and would thus the LED pulse would be effectively similar to a single turnover flash distributed in time over the population of centers. Variations and refinements of the original two-quantum model have been advanced based upon alternative techniques for illumination and O_2_ detection during photoactivation (Miller and Brudvig, 1989; Meunier et al., 1995; Ananyev and Dismukes, 1996b; Zaltsman et al., 1997; Hwang and Burnap, 2005). The multiflash experiments of Hwang and Burnap (2005) using staggered Xe single turnover flashes revealed a new kinetic intermediate, more rapid rearrangement, although where it is in the sequence could not be established owing to high miss factor (low quantum efficiency) and the associated de-phasing of the assembly during the flash induced assembly process.

### Are the complicated “two-quantum” kinetics of photoactivation an artifact of *in vitro* experimental procedures?

Many of the insights into the mechanism, including the nature of the cofactor requirements, were from experiments performed *in vitro* using biochemical techniques, including detergent solublization, that yield simplified PSII preparations. Such preparations allowed a range of information from the better definition of the affinity constants for the cofactors (Tamura and Cheniae, 1986, 1987; Miller and Brudvig, 1989; Tamura et al., 1989; Ananyev and Dismukes, 1996a,b, 1997; Zaltsman et al., 1997; Baranov et al., 2000, 2004) to the comparative efficiency of artificial electron acceptors (Miyao and Inoue, 1991; Miyaotokutomi and Inoue, 1992). However, the most efficient *in vitro* protocols explicitly involve the remove of extrinsic proteins or involve procedures that would also cause the loss of extrinsic proteins, although the authors may not have evaluated the degree to which this loss may have occurred. Importantly, the simplified preparations also likely lack the multiple assembly factors that are now identified. Since photoactivation is a low quantum yield process, even *in vivo*, it has been important for technical reasons to maximize the *in vitro* efficiency to estimate its kinetic parameters. For example, a Mn^2+^ concentration dependence assays require modest increments in yield, which can only be experimentally distinguished if the procedures provide materials with sufficiently high rates of O_2_ evolution to allow discrimination beyond to envelope of experimental errors. Thus, in the development of the procedures, removal of the extrinsic proteins provided researchers with a system that satisfied these requirements. The mutational loss of the extrinsic proteins increases the quantum efficiency of photoactivation (Burnap et al., 1996; Shen et al., 1998) as do mutations that weaken the binding of the extrinsic proteins (Qian et al., 1997, 1999). This appears to be due greater access of the Mn^2+^ ions to their site of photooxidation on the donor side of PSII (Chu et al., 1994a). However, this begs the question of how much the “natural” physiological kinetics are distorted by the loss of the extrinsic proteins and what other factors that might be removed or inactivated in the process. Indeed, it is almost certain that important assembly factors may have been absent in many of the defining photoactivation experiments. From this standpoint, it is clear that the *in vitro* photoactivation experiments have been decidedly non-physiological. Then what are the implications for the kinetics that have defined the mechanism to date? Here it is worth noting that the basic two quantum mechanism was first discovered using intact cells and chloroplasts. This includes experiments in samples that were not extracted with chemical reductant to remove the Mn_4_O_5_Ca. For example, these include using intact chloroplasts from leaves grown under intermittent light to promote de-etiolation, but remaining un-photoactivated (Ono and Inoue, 1982, 1983) and cyanobacterial cells grown under conditions of Mn-deficiency, and dark grown (with glucose) *Chlorella* cells (Cheniae and Martin, 1973). These samples are likely have the full complement of extrinsic proteins and assembly factors. Similarly, the extraction of whole cyanobacterial cells and chloroplasts with the hydroxylamine probably preserves many if not most assembly factors as operational. This probably explains the fact that nearly 100% of PSII centers become reactivated by photoactivation in these more intact preparations, but *in vitro* preparations typically have considerably lower total yields. The more “physiological” preparations still exhibit (1) low quantum efficiency and (2) the requirement for optimal flash spacing. However, inspection of data involving dark grown *Chlorella* indicates that photoactivation occurs more quickly, although optimal flash spacing is still required. Clearly, the role of assembly factors needs to be further pursued. One might imagine in this case that a closely associated assembly factor, like PratA, provides Mn^2+^ ions at critical times during the formation of intermediates and thereby mitigates potential losses due to intermediate decay. Except for one instance where the Psb27 mutant was analyzed (Roose and Pakrasi, 2007), a careful side-by-side comparisons of photoactivation in mutants and wild-type remain to be performed.

### High affinity binding site

Biochemical preparations of PSII that have been depleted of their Mn_4_O_5_Ca have been used to test the binding affinity for Mn^2+^. The principal finding is that a single binding site, termed the high affinity site, dominates the kinetics. The dissociation constant for Mn^2+^ at this site is estimated to be in the range of 0.1–2 μM (Hsu et al., 1987; Diner, 2001) and is strongly pH dependent (pK_*a*_ 6–7) (Ono and Mino, 1999). As described below, these are accurate, but essentially, non-equilibrium assays. In one of the few examples of an estimate of the true equilibrium binding constant, a significantly higher value of 40–50 μM was estimated. In this case, binding was allowed to occur in the dark and the samples were frozen to −20°C, where diffusion was eliminated and an EPR binding photoxoidation signal could be detected, thereby giving a “snapshot” of the amount of photooxidizable Mn^2+^ bound at equilibrium (Tyryshkin et al., 2006). Notably, the binding affinity was found to be independent of the binding of Ca^2+^. Almost all other assays have been performed utilizing the ability of Mn^2+^ to donate electrons to photochemically generated ${\text{Y}}_{\text{Z}}{}^{•}$. Therefore, the high affinity site has been largely defined biochemically based upon the combination of affinity *and* the ability to be photooxidized by ${\text{Y}}_{\text{Z}}{}^{•}$ during charge separation, rather that equilibrium binding assays alone (see older review Debus, 1992, for still up-to-date discussion). -This high affinity/efficient oxidation site remains intact in the mutant without processing of D1 carboxy terminus (Nixon et al., 1992), although subtle differences in the affinity characteristics of these mutants are observed when the biphasic kinetics of the binding/oxidation are fully taken into account (Cohen et al., 2007). On the other hand, the access of Mn^2+^ to this site is significantly increased in the carboxyterminal processing mutants as well as mutants lacking extrinsic proteins (Chu et al., 1994a; Semin et al., 2015). Mutagenesis of D1-Asp170 has shown that the residue clearly has the strongest effect upon on the affinity of Mn^2+^ and the ability to assemble a fully functional Mn_4_CaO_5_ cluster (Boerner et al., 1992; Diner and Nixon, 1992; Nixon and Diner, 1992; Chu et al., 1994b; Whitelegge et al., 1995; Campbell et al., 2000; Cohen et al., 2007). This is consistent with the crystal structures of PSII (Umena et al., 2011; Suga et al., 2015), which have shown that Asp170 is a ligand to the Mn(4) of assembled (intact) Mn_4_CaO_5_ cluster (Figure 2). Notably, the other carboxyl O of the D1-Asp170 side chain provides a monodentate ligand to the adjacent Ca^2+^ ion of the assembled cluster, which probably relates to the Ca^2+^ requirement photoactivation, as discussed below. Other residues, notably the other main amino acid ligand to Mn(4), D1-Glu333 affect the affinity characteristics of the high affinity binding site, but none as decisively as mutations of D1-Asp170 (Cohen et al., 2007).

D1-Glu333 is a ligand to Mn(4) presumptive the first photo-oxidized Mn^2+^ at the high affinity binding site (Umena et al., 2011). In all mutants of Glu333, substantial fractions of PSII complexes lack photooxidizable Mn ions *in vivo* (Chu et al., 1995), showing that Glu333 influences the assembly or stability of the Mn_4_CaO_5_ cluster. Nevertheless, mutations of Glu333 do not display the large changes in Mn^2+^ affinity compared to D1-Asp170 mutations, at least as measured using single turn-over methods to assay affinity (Nixon and Diner, 1994; Cohen et al., 2007). One possibility to accommodate these observations is that Glu333 provides some coordination of Mn^2+^ ions at the high affinity site, but play an even greater role in the subsequent assembly process. Pulsed electron-electron double resonance (PELDOR) experiments have recently provided the evidence to support this hypothesis that the high-affinity Mn^2+^ site is located at the position denoted by Mn(4) in the crystal structure and the first photooxidized Mn^2+^ bound to the apo-WOC is coordinated with axial ligands Asp170 and Glu333 in the D1 protein (Asada and Mino, 2015). These results thus substantiate and extend the initial assessments of the high affinity site based upon site-directed mutagenesis, yet deepen the puzzle about the seeming modest influence this axial ligand has to the affinity/photooxidation characteristics of the site. For the C-terminal residues D1-Ala344, neither mutations in the C-terminal region of D1 nor the processing of the C-terminal extension (Ala344stop and Ser345Pro mutants) has a large influence on the ability to bind and oxidize the first Mn^2+^ in the assembly of the cluster (Nixon et al., 1992). These observations indicate that these C-terminal residues do not participate in the coordination of the first bound Mn, though they certainly must contribute to the coordination of those bound later on in the assembly process (Diner, 2001). In the recent crystal structure of PS II, His337 residue is sufficiently close to Mn_4_CaO_5_ cluster and engage in H-bonding interactions with the μ_3_-oxo bridge connecting Mn(1), Mn(2), and Mn(3) (Umena et al., 2011).

### Trapping intermediates of photoassembly

Britt and coworkers (Campbell et al., 2000) provided the first direct EPR spectral evidence for the initial photooxidized intermediate formed at the high affinity site in *Synechocystis* 6803 PSII core complexes. Conventional perpendicular-mode EPR in X-band is used to detect spin transitions in half integer spin systems which satisfy the selection rules ΔM_s_ = ± 1. Accordingly, the first light induced Mn^3+^ species, due to the integer spin S = 2 of Mn^3+^, is an EPR-silent species for perpendicular polarization EPR spectroscopy at X-band frequencies. X-band parallel polarization EPR spectroscopy, however, can be used to investigate integral spin systems with S ≥ 1 where the spin transitions satisfy the selection rules ΔM_s_ = ± 2 and higher. This latter technique is therefore well-suited to examine the coordination environment of this Mn^3+^ intermediate (high spin S = 2). A six-line signal with a hyperfine splitting of ~45 G that was only visible in parallel mode. This signal clearly arises from Mn^3+^ as it closely resembles that observed for Mn^3+^ in superoxide dismutase (Campbell et al., 1999). The parallel mode EPR spectrum of this photooxidation species consists of six well-resolved transitions split by a relatively small ^55^Mn hyperfine coupling (44 G). The Mn^3+^ parallel mode EPR signal gives an axial zero-field splitting value of D ≈−2.5 cm^−1^ and a rhombic zero-field splitting value of |E| ≈ 0.269 cm^−1^. The negative D value for this d^4^ ion is indicative of either an octahedral Mn^3+^ geometry or a five-coordinate square-pyramidal Mn^3+^ geometry. In contrast to wild-type, a different parallel polarization EPR signal of Mn^3+^ ion without a resolved hyperfine structure was observed in Asp170His mutant, suggesting a modified coordination environment of Mn^3+^. In the case of Asp170Glu mutant, instead of a parallel mode Mn^3+^ signal, a perpendicular mode signal generated by Mn^4+^ ion was detectable (Campbell et al., 2000), which indicates an impact of glutamate on the redox property of the photo-oxidized Mn^2+^ ion. As noted previously (Hoganson et al., 1989), coordination by oxo anions as would have effect of lowering the redox potential of the Mn^2+^ ion into the range that the oxidizing potential of ${\text{Y}}_{\text{Z}}{}^{•}$.

The weak EPR signal found by Dismukes et al. in the dark apo-PSII samples upon binding of Mn^2+^ in the absence of Ca^2+^ is characterized by six-line ^55^Mn hyperfine structure and g_*effe*_ = 8.3, which indicates a high-spin electronic ground state (S = 5/2) of Mn^2+^ bound in a low-symmetry environment (Ananyev and Dismukes, 1997). This signal is likely arise from a Mn^2+^ bound in the high-affinity site. Dismukes et al. (Ananyev et al., 1999) later suggested using competitive inhibition studies that the first species that initiated photoactivation (Ananyev et al., 1999) is hydroxide of Mn^2+^, [MnOH]^+^, bound to the apo-WOC at high affinity site. Subsequent work by the same group would provide evidence that the hydroxide formation was modulated by Ca^2+^ (Tyryshkin et al., 2006).

### Role of Ca^2+^ and the Ca^2+^ bound intermediate

Ca^2+^ is an indispensable cofactor of the water-splitting Mn_4_CaO_5_ cluster. As noted in the previous section, biophysical studies attempting to trap early intermediates showed that Ca^2+^ exerts pronounced, and possibly physiologically significant effects upon the structure of Mn ions undergoing photooxidation at the high affinity site. However, from the biochemical perspective, the roles of Ca^2+^ ion in the process of photoactivation *initially* appeared contradictory: A requirement for Ca^2+^ in photoactivation was also noted using cyanobacterial preparations (Pistorius and Schmid, 1984). Ono and Inoue (1983) proposed that photoactivation occurs in one stage with Ca^2+^ essential for the assembly process itself using isolated intact chloroplasts depleted of Mn. According to the one-stage model the first-order rate constant for the assembly of O_2_-evolving centers is dependent on the extent of occupancy of both Mn^2+^ and Ca^2+^ bound to their specific binding sites during photoactivation. Later experiments seemed to indicate that the Ca^2+^-binding site is “created” during the photoassembly (Shinohara et al., 1992). For example, Tamura and Cheniae (1987) found that only light and Mn^2+^ were essential for Mn re-ligation to the apo-WOC-PSII, but Ca^2+^ addition was required for maximal expression of water oxidation activity by the photoligated Mn. In other words, it appeared that Ca^2+^ was not required for proper assembly, but was needed as a cofactor that readily diffused into its site of action after the assembly of the Mn cluster was completed and, once in place, activated its catalytic function. However, this conclusion was later modified to account for the complicating effects of the artificial electron acceptor used in the assay (Chen et al., 1995a). Ultimately, it was thus concluded that Ca^2+^ is indeed absolutely required *during* the assembly of functional clusters, not simply being added in after assembly (Chen et al., 1995a). The same work provides what may be another important clue about the role of Ca^2+^. It was found that photoactivation of PSII membranes in the absence of Ca^2+^ led to the formation of inactive PSII with more than four Mn ion per PSII center (5–10 non-functional Mn per PSII). Thus, when Ca^2+^ is left out of the photoactivation medium, binding and photooxidation of many more Mn^2+^ to the apo-WOC-PSII protein occurs, but no O_2_ evolution activity is observable (Chen et al., 1995a). Manganese bound in this way could be released with reductant indicating that it was bound oxidatively, but it clearly cannot bind to specific protein binding sites. Instead, probably resembles amorphous oxides which are multinucleate metal-oxo deposits produced by inorganic processes (Sauer and Yachandra, 2002). This suggests that one role for Ca^2+^ is to guide assembly or simply block Mn^2+^ oxidation at the Ca^2+^ site, which prevents “inappropriately assembled” Mn (Chen et al., 1995a). Interestingly, mutants that are defective in processing the D1 carboxy terminus also seem to assemble centers with excess Mn (Seibert et al., 1989). Recent X-ray crystallographic studies (Umena et al., 2011) provides a structural explanation for the Seibert result. The PSII structure reveals that mature D1 C-terminal residue Ala344 ligate the Ca^2+^ and Mn(2) of Mn_4_CaO_5_ cluster, thus without the availability of the mature C-terminus Ala344, Ca^2+^ cannot bind and the destructive photoligation of Mn^2+^ to inappropriate sites can proceed. Additionally, competition between Ca^2+^ and Mn^2+^ for each other's binding sites has been indicated by many studies (Cheniae and Martin, 1971b; Radmer and Cheniae, 1971; Ono and Inoue, 1983; Tamura and Cheniae, 1987; Miller and Brudvig, 1989; Chen et al., 1995a,b; Ananyev and Dismukes, 1996a; Zaltsman et al., 1997). Since Sr^2+^ can substitute of Ca^2+^ in PSII *in vivo*, albeit with impaired H_2_O-oxidation activity (Boussac et al., 2004), it would be interesting to see how photoactivation occurs with this substitution. However, only limited information is currently available (Ananyev et al., 2001).

The effect of Ca^2+^ on the formation of the first photoactivation intermediate, corresponding to a photooxidized mononuclear Mn^3+^ species bound to apo-WOC-PSII, was investigated by EPR spectroscopy (Tyryshkin et al., 2006). In the absence of Ca^2+^, the Mn^3+^ species was found to be generated as two forms in a pH-dependent equilibrium: an EPR-invisible low-pH form and an EPR-visible high-pH form. Note, these spectra of Mn^3+^ species were acquired in parallel mode and EPR invisible vs. visible is attributable to changes in the influences in ligand environment rather than the spin state selection rules noted above. The conversion between the visible and invisible forms occurs by deprotonation of an ionizable ligand bound to Mn^3+^, postulated to be a H_2_O molecule: [Mn^3+^(OH_2_)]↔[Mn^3+^(OH^−^)]. The EPR-visible high-pH form exhibits a strong pH effect (pH 6.5–9) on Mn^3+^ spectral parameters, including the rhombicity (δ) derived from center field position (g_*eff*_), the ^55^Mn hyperfine coupling (A_*Z*_), and the signal intensity. A pH-induced protein conformational change was proposed to account for the observed significant changes in the symmetry of the ligand field at the Mn^3+^ site. On the other hand, the EPR-detectable Mn^3+^ induced in the presence of Ca^2+^, exhibits a greatly weakened pH dependence of its ligand-field symmetry with reduced variation of rhombicity δ and ^55^Mn hyperfine coupling A_*Z*_in the pH range of 6.5–9.0. Moreover, the addition of Ca^2+^ moves both g_*eff*_ and A_*Z*_ to a range of values observed at alkaline pH ≥ 9 without added Ca^2+^, indicating that Ca^2+^ binding exerts an influence on the coordination shell of Mn^3+^ species equivalent to the alkaline pH effect in the absence of Ca^2+^. Therefore, it was proposed that Ca^2+^ binding induces a second ionization of the bridging hydroxo ligand bound to Mn^3+^ resulting in the formation of a bridging oxide ion ([Mn^3+^(OH^−^)-Ca^2+^] ↔ [Mn^3+^(O^2−^)-Ca^2+^]). The proton ionization of the water ligand is postulated to be controlled by a nearby base B^−^, which serves as an immediate proton acceptor with a p*K*_*a*_ that depends upon the occupancy of the Ca^2+^ effector site. Looking at the current crystal structure and assuming a similar, although almost certainly not identical, spatial configuration of the Ca and Mn, the inferred oxo bridge would join these ions with D1-Asp170, Glu333 and the carboxyl terminus in proximity of one another.

### Other inorganic cofactors

There is a long debated role of inorganic carbon in photosynthetic water oxidation. Recently, it has been demonstrate that bicarbonate (${\text{HCO}}_{3}^{-}$) can act as a mobile acceptor and transporter of protons produced by photosynthetic water oxidation PSII (Koroidov et al., 2014). Bicarbonate also seem to have an impact on photoassembly of Mn_4_CaO_5_ cluster (reviewed in Dasgupta et al., 2008). The proposed roles of bicarbonate in facilitating assembly of Mn_4_CaO_5_ cluster during PSII repair include acceleration of the binding and photooxidation of the first Mn^2+^ at the high affinity Mn site, putatively by increase the location concentration of Mn^2+^ and even direct ligation to Mn^2+^ (Baranov et al., 2000, 2004; Dasgupta et al., 2007). Bicarbonate has been found not an essential constituent of the WOC of PSII based on the most recent PSII crystal structure (Umena et al., 2011), making the direct ligation seem unlikely. However, this does not necessarily mean that the possibility of a weakly bound ${\text{HCO}}_{3}^{-}$ at the donor side affecting the PSII repair has been excluded. It is also important to note that high concentrations of Cl^−^ also enhance photoactivation *in vitro*, but this appears to be an effect distinct from the known effects on the activation of the photosynthetic water oxidation catalytic activity. Instead, it more likely relates to the stabilization of the Mn_4_CaO_5_ in the absence of the extrinsic proteins in the studied photoactivation reactions (Miyao and Murata, 1985; Miyaotokutomi and Inoue, 1992). The catalytic activation properties of Cl^−^ are likely to be exerted indirectly upon water and/or proton movement during H_2_O-oxidation given it binding locations in the second ligation sphere of the assembled Mn_4_O_5_Ca.

### Possible models of assembly

Although the two-quantum model remains solidly at the foundation of our understanding of photoactivation, the molecular mechanisms that give rise to these kinetic features remain almost completely unresolved. A crucial question is what molecular processes gives rise to the so-called dark rearrangement. As discussed, consistent models would require occupancy of the high affinity site by Mn^2+^ and the presence Ca^2+^ at a nearby site, and the Ca^2+^ probably needs to be present during the initial photooxidation of the Mn^2+^. This first photooxidation (A⇒B) likely occurs with high quantum efficiency, yet the photooxidation of the second Mn^2+^ (C⇒D) occurring after the rearrangement occurs with low quantum efficiency. There are essentially two general alternative hypotheses accounting for the low quantum yield of C⇒D that depend, in part, on the nature of the rearrangement. First, the rearrangement after A⇒B forms a binding site for the second Mn^2+^ that is not optimal for electron transfer to ${\text{Y}}_{\text{Z}}{}^{•}$, perhaps because it is further away from the high affinity site and because of this, charge recombination effectively competes with the low probability of photooxidation of the second Mn^2+^ at the new site. Please note that the charge recombination may actually be the ‘rearrangement’ and could be important for removing mis-assembled clusters (Hwang et al., 2007). Second, the rearrangement is again a slow process, but this time leads to formation of efficient site for Mn^2+^ photooxidation, but its initial product, the first Mn^3+^ fails to convert efficiently into a stable chemical product. This could happen if, for example, a requisite oxo bridge forms inefficiently and the new Mn^3+^ diffuses away or is re-reduced. Based upon the finding that the C-terminus of D1 is already in a conformation close to the final configuration in the fully assembled enzyme (Cohen et al., 2007), then the dark rearrangement is unlikely to be a major rearrangement of the D1 polypeptide backbone. With this constraint, we imagine these two alternative assembly mechanisms as follows. One alternative is that the slow rearrangement corresponds to slow oxo bridge formation chemistry that is kinetically very sluggish, occurring with the rate constant k_*R*_ after the initial photooxidation. Further, once the first Mn^3+^ is produced at the high affinity site, the new binding site for the second Mn^2+^ is at a more distal site where the quantum yield of its photooxidation lower. In this case, the first Mn^3+^ at the high affinity site is already engaged oxo linkage with the Ca^2+^ (Tyryshkin et al., 2006) and one of its other coordination positions occupied by a water must deprotonate to form a stable linkage with the second Mn^2+^. According to this hypothesis, the slow formation of the second oxo corresponds to k_*R*_ and the low quantum yield of C⇒D is due to the more distal location from ${\text{Y}}_{\text{Z}}{}^{•}$. However, one problem with this model is that each of the subsequent two Mn additions would seem to have to also occur with low quantum yields, since the high affinity site remains occupied. However, these later photoligations are thought to occur with high quantum efficiency, although the evidence even for that remains sparse. A second alternative to a primarily protein rearrangement is a rearrangement of the ions. As mentioned above, the high affinity site has been largely defined biochemically based upon the combination of affinity *and* the ability to be photooxidized by ${\text{Y}}_{\text{Z}}{}^{•}$ during charge separation. Perhaps the high affinity site remains the site of oxidation for each of the photooxidations for the assembly reactions and the resultant Mn^3+^ ions migrate to their final locations. In this model, the relocation of the Mn ion, vacating the high affinity site, and its coordination into its new site accounts for the “dark rearrangement.” However, the dissociation of the Mn^3+^ dissociates from its initial binding and oxidation site presents a problem because of the likely increase in ligand field stabilization energy. This would not be a problem for Mn^2+^, which has no associated LFSE. On the other hand, Mn^3+^ should bind with a substantially higher affinity owing to the acquisition of LFSE in the higher oxidation state. There may be meahisms to alleviate this problem such as a redox disproportionation of bound Mn^3+^-Mn^2+^ to (Mn^2+^-Mn^3+^ so the original Mn^3+^ is now weakly bound (no LFSE) and can exchange to another site. The EPR observations consistent with oxo bridge formation Ca^2+^ ion accompanying the photooxidation of Mn^2+^ ions at the high affinity site suggest a templating function for Ca^2+^ during assembly. Based upon the crystal structure, the Ca^2+^ would be captured between D1-Asp170 and the carboxyterminal carboxylate of the D1 protein. As noted, there is good evidence that Ca^2+^ binding induces a second ionization of the bridging hydroxo ligand bound to Mn^3+^ resulting in the formation of a bridging oxide ion ([Mn^3+^(OH^−^)-Ca^2+^] ↔ [Mn^3+^(O^2−^)-Ca^2+^]) (Tyryshkin et al., 2006). If the Mn^3+^ leaves the high affinity site, then Ca^2+^ may tether the new Mn with an oxo (or hydroxo) bridge and facilitate the movement to the next site. Moreover, the water molecules coordinated Ca^2+^ may be provided as the substrate for additional μ-oxo bridge formation. In this way, Ca^2+^ functions in a manner similar to proposals for its role in catalytic water oxidation and substrate exchange though the positioning of coordinated waters (Vrettos et al., 2001; Hillier and Wydrzynski, 2008; Rappaport et al., 2011; Cox and Messinger, 2013). According to electrostatic calculations, there are substantial differences in the redox potentials for each of the four spatially distributed Mn ions of the assembled Mn_4_CaO_5_ cluster due to their specific coordination and large electrostatic environments (Amin et al., 2015). Based upon these electrostatic calculations of Amin et al. (2015), it is likely that Mn^3+^ ions are thermodynamically more stable at coordination positions elsewhere within the partially assembled cluster in comparison to their primary site of oxidation at the high affinity site. It also fits with a variety of biochemical studies showing that one pair of Mn ions, presumably a binuclear di-μ-oxo bridged unit, in the assembled cluster is more stable and has different accessibility to external reductants than the other pair (Frankel and Bricker, 1989; Mei and Yocum, 1991, 1992; Riggs et al., 1992). In this model, the rearrangement time may correspond to the relocation of the first Mn^3+^ ion to exit the high affinity site and relocate to another site, perhaps guided via a nascent oxo with the Ca^2+^.

## Author contributions

HB researched the topic and wrote the initial draft of the manuscript. RB added materialand made figures, performed additional research and edited the draft.

## Funding

The work was generously funded by a grant form the National Science Foundation (MCB-1244586).

### Conflict of interest statement

The authors declare that the research was conducted in the absence of any commercial or financial relationships that could be construed as a potential conflict of interest.
